# Fidelity

**Published:** 1879-09

**Authors:** 


### FIDELITY.

CONSCIENTIOUS fidelity, in the performance of duty owed to others, is the great want of the age. A very large share of all the accidents, and sickness, and disappointments, and sorrows, and heart-breakings of life can be traced to the want of doing things right and well. A switch-tender neglects his duty—a railway train is driven from the track, and a hundred broken bones and fifty lives lost is the record of the next half minute. An engineer is carelessly talking to a lounger on a beautiful Sabbath morning at the South Ferry—the boiler explodes, and more than one hundred persons, in a second's time, are ushered into eternity. The caulker omits to stop an auger hole—the ship goes down in mid-ocean, and no human being ever returns to tell the sad tale. A bank cashier does not scrutinize a signature with sufficient minuteness—money is paid out on a forged name, the institution fails, and widows and orphan children are beggared for life.

Large penalties, swift and sure, will but partially secure a more careful discharge of duty. The only radical remedy, universal and unfailing, is to be found in the prevalence of the sentiment, "Thou, God, seest me."

